# Thermal, Mechanical, Morphological, and Piezoresistive Properties of Poly(ethylene-co-methacrylic acid) (EMAA) with Carbon Nanotubes and Expanded Graphite

**DOI:** 10.3390/nano15130994

**Published:** 2025-06-26

**Authors:** Francesca Aliberti, Luigi Vertuccio, Raffaele Longo, Andrea Sorrentino, Roberto Pantani, Liberata Guadagno, Marialuigia Raimondo

**Affiliations:** 1 Department of Industrial Engineering, University of Salerno, Via Giovanni Paolo II, 84084 Fisciano, Italy; rlongo@unisa.it (R.L.); rpantani@unisa.it (R.P.); lguadagno@unisa.it (L.G.); 2 Department of Engineering, University of Campania “Luigi Vanvitelli”, Via Roma 29, 81031 Aversa, Italy; luigi.vertuccio@unicampania.it; 3 Institute for Polymers, Composites, and Biomaterials (IPCB-CNR), Via Previati n. 1/E, 23900 Lecco, Italy; andrea.sorrentino@cnr.it

**Keywords:** carbon nanotubes, expanded graphite, strain sensitivity, soft nanocomposites

## Abstract

This paper presents a comparative study examining the effects of carbon nanotubes (CNTs) and expanded graphite (EG) on the thermal, mechanical, morphological, electrical, and piezoresistive properties of poly(ethylene-co-methacrylic acid) (EMAA) nanocomposites. To this end, different amounts of carbonaceous fillers (EG and CNTs separately) were added to the EMAA thermoplastic matrix, and the relative electrical percolation thresholds (EPTs) were determined. The effect of filler concentration on thermo-oxidative degradation and the EMAA crystallinity was investigated via thermogravimetric analysis (TGA) and differential scanning calorimetry (DSC), respectively. Dynamic mechanical analysis (DMA) demonstrated that both fillers enhance the Young’s and storage moduli, as well as the glass transition temperature, with a greater improvement for the bidimensional nanofiller, most likely due to the cumulative effect of more extensive EG-matrix interactions. In tensile tests, a very relevant difference was detected in the Gauge Factor (G.F.) and the elongation at break of the two typologies of nanocomposites. The G.F. of EMAA 10% CNT and EMAA 15% EG were found to be 0.5 ± 0.08 and 165 ± 14, respectively, while elongation at break was about 68% for EMAA 10% CNT and 8% for EMAA 15% EG. Emission Scanning Electron Microscopy (FESEM) and Tunneling Atomic Force Microscopy (TUNA) have contributed to explaining the differences between EG- and CNT-based nanocomposites from a morphological point of view, underlying the pivotal role of the filler aspect ratio and its structural features in determining different mechanical and piezoresistive performance. The comprehensive analysis of EMAA-EG and EMAA-CNT nanocomposites provides a guide for selecting the best self-sensing system for the specific application. More specifically, EMAA-CNT nanocomposites with high elongation at break and lower sensitivity to small strains are suitable for movement sensors in the soft robotic field, where high deformation has to be detected. On the other hand, the high sensitivity at a low strain of EMAA-EG systems makes them suitable for integrated sensors in more rigid composite structures, such as aeronautical and automotive components or wind turbines.

## 1. Introduction

Poly(ethylene-co-methacrylic acid) (PEMAA) is a thermoplastic soft material available on the market in the form of copolymers and ionomers. Its chemical structure is characterized by the presence of carboxyl groups along the backbone of the macromolecules. More specifically, EMAA has a block structure composed of segments of poly(ethylene) (E) and polymethacrylic acid (MAA). The characteristic elasticity of EMAA is due to the E-segments that favor a partial recovery of the initial shape, while the MAA segments containing carboxylic groups establish intermolecular chemical interaction (i.e., hydrogen bonds). In the case of ionomers, the acid groups are partially neutralized by metallic ions such as Sodium (EMAA_Na) or Zinc (EMAA_Zn) ions, which are responsible for ionic cross-links [1]. EMAA copolymer and its ionomers have attracted a lot of interest in the research field because of their peculiar self-healing and shape memory properties [2,3]. The mobility of the E-segment, coupled with the strong attraction of MAA segments, allows the reduction in delamination of composites [4], to repair damage and improve energy dissipation during impact [5]. This is the reason why EMAA has been widely applied as a self-healing material in epoxy resins [6,7,8,9] and in glass/carbon fiber composites [7,9,10,11,12,13] to mitigate the costly maintenance of aeronautical vehicles, facilitate the repair of difficult-to-access structures (e.g., wind-turbine blades), and reduce part replacement. More recently, Snyder et al. [14] used the Fused Filament Fabrication (FFF) process to locally deposit beads of EMAA into a glass fiber and carbon fiber-reinforced panel to impart self-healing function to the final composite structure, demonstrating the possibility of processing this material with Additive Manufacturing (AM) technologies. EMAA has been revealed to be a valuable and advantageous component that is also blended with other thermoplastic polymers. Blending thermoplastic materials is a common practice in the industrial and research fields to combine the benefits of two or more materials [15]. As proof of this, EMAA has been blended with polyethylene (PE) to improve adhesion properties and obtain a matte surface for packaging applications [16]. It has been demonstrated that only 3 wt% of EMAA copolymer is sufficient to make a 3:1 mass ratio of polyethylene terephthalate (PET) bottles and polyethylene bags compatible with the recycling of plastic wastes [17]. The poly(ε-caprolactone)/EMAA-Zn blend has been studied to merge PCL diffusion with thermo-responsive shape memory, ionic interactions, and supramolecular bonds of EMAA-Zn to obtain a final material of healing efficiencies between 90% and 96% [18].

However, EMAA could be a good candidate for strain sensor applications due to its high stretchability. To the author’s knowledge, only a few papers in the literature focus on EMAA nanocomposites for self-sensing applications. Using nanoparticles is one of the most effective strategies for adding self-sensing function to EMAA polymer. Carbon nanotube (CNT) [19,20,21], expanded graphite (EG) [22], short carbon fibers [23,24], carbon black [25,26], and metallic and ceramic nanoparticles [27,28,29] are the most used filler for improving the electrical, thermal, and mechanical properties of the thermoplastic matrix and confer smart functions to the resulting nanocomposite materials [30,31]. Among them, CNTs and EG are the most interesting due to their good compromise between overall performance (thermal, mechanical, and electrical) and cost, availability, and ease of processing from an industrial point of view [32]. Moreover, it is extremely riveting to investigate the effect of the shape and aspect ratio of these nanofillers on the final properties of the nanocomposites. In fact, CNTs and EG differ in shape and structural features. The classification of carbon nanotubes (CNTs) as 1D nanofillers and expanded graphite (EG) sheets as 2D nanofillers is based on their structural geometry and dimensional confinement. The cylindrical, tube-like shape of CNTs gives them a high aspect ratio, meaning their length is significantly greater than their diameter on the nanometric scale. This one-dimensionality affects their electronic, mechanical, and optical properties, making them ideal for reinforcement in polymer composites. EG sheets consist of exfoliated layers of graphene-like structures with nanometric thickness but micrometer-scale lateral dimensions. Their two-dimensional nature enables excellent electrical conductivity, thermal stability, and significant surface area interactions. Their differences play a crucial role in composite applications, whether enhancing mechanical strength, electrical conductivity, or strain sensing.

Basuli et al. [33,34] were among the first researchers interested in EMAA-CNT nanocomposites. The authors focused their attention on morphological, thermal, and mechanical characterization. Cohen et al. [35] performed an investigation a few years later, trying to lower the electrical percolation threshold by non-covalent compatibilization of the EMAA matrix and CNTs with 4-(aminomethyl)pyridine. The applications mentioned in their work include antistatic packaging, electromagnetic interference shielding films, conductive inks, paints, and a conductive coating applied to polyolefin matrices. Another example of EMAA nanocomposite is given by Tita et al. [36], who exploited zirconate titanate as a piezoelectric active filler (PZT) to develop reliable piezoelectric strain sensors applicable over an extensive temperature range, from liquid nitrogen temperature to the cure temperature of high-performing polymer matrix composite materials, for in situ health monitoring of aircraft components. Lastly, the only example of piezoresistive characterization of the EMAA-CNT system is provided by Hia et al. [37]. In their work, the authors utilized fused filament fabrication (FFF), an additive manufacturing (AM) technology, to customize and directly deposit an EMAA-CNT-based piezoresistive sensor onto a glass fiber composite for structural health monitoring (SHM) applications, achieving minimal sensor hysteresis after 20 flexural load–unload cycles.

Regarding the incorporation of EG filler in the EMAA matrix, Cerezo et al. [38] studied the effect of various graphite forms on the crystal structure, thermal properties, thermo-mechanical behavior, and dielectric properties of the above-mentioned matrix. However, in the literature sources, there is no direct reference to the piezoresistive response of EMAA-EG nanocomposites. As a consequence of this, there is also a lack of comparative studies of the effect of carbon nanotubes (CNTs) and expanded graphite (EG) on the thermal, mechanical, morphological, electrical, and piezoresistive properties of EMAA nanocomposites. In this context, the present paper aims to address the current literature’s lack of coverage, both in terms of the study on the piezoresistive performance of EMAA-EG nanocomposites for strain monitoring and in terms of the comparison of EMAA-EG systems with those based on CNTs. This paper, therefore, represents the first successful attempt to highlight and investigate the differences and commonalities between these two nanofilled systems through an extensive and in-depth correlation of thermal, mechanical, morphological, electrical, and piezoresistive results.

## 2. Materials and Methods

EMAA copolymer (CAS#9010-77-9) has been provided by Scientific Polymer Products, Inc. (Sp2) (Ontario, NY, USA). Carbon nanotubes (CNTs) and the expanded graphite (EG) used in the present paper were supplied by ARKEMA (GRAPHISTRENGTH C100) and by Superior Graphite Co (Chicago, IL, USA), respectively. The authors have calculated the aspect ratio of these two fillers in previous work; it is around 1000 for the CNTs and between 1128 and 3541 for EG [39].

EMAA nanocomposite blends were obtained by mixing overnight dried pristine EMAA copolymer in the form of pellets with nanofiller powder in a twin counter-rotating internal mixer (Rheomix 600 Haake, Germany) connected to a control unit (Haake PolyLab QC). The mixing temperature, rotation speed, and mixing time were 150 °C, 50 rpm, and 10 min, respectively. These process conditions are common to all prepared blends. Then, nanocomposite materials are obtained in sheets approximately 1 mm thick via the compression molding process, for which a Carver press (Wabash, IN, USA) was used. Thermogravimetric analysis (TGA), differential scanning calorimetry (DSC), and dynamic mechanical analysis (DMA) were performed using the equipment and methods described in Table 1.

Before the FESEM investigation, the samples were subjected to a procedure to remove part of the polymeric matrix using an oxidizing solution (etching solution). More precisely, the etching reagent was prepared by stirring 1.0 g of potassium permanganate in a solution of 95 mL of sulfuric acid (95–97%) and 48 mL of orthophosphoric acid (85%). The nanocomposites were immersed in the fresh etching reagent at room temperature and held under agitation for 36 h. Subsequent washings were performed using a cold mixture of two parts by volume of concentrated sulfuric acid and seven parts of water. Afterward, the samples were washed again with 30% aqueous hydrogen peroxide to remove any manganese dioxide and, finally, with distilled water and kept under vacuum for 5 days before being subjected to morphological analysis. The etching treatment was also used for the TUNA investigation. TUNA images were elaborated using the Bruker software Nanoscope Analysis 1.80 (BuildR1.126200). Electrical conductivity was measured for all the experimentally prepared EMAA nanocomposites, using a two-probe method on specimens with a rectangular shape. The electrical contacts were realized on the sample surface with silver paint (RS 196-3600, RS PRO, Corby, UK). The measurements were carried out using an electrometer (Keithley 6517A, Keithley Instruments, Cleveland, OH, USA), which acted as both a power supply and an ammeter. The contact resistance has been considered negligible since the measured electrical resistance was in the order of kΩ. The power supply provides different voltage values within the range of 0 to 25 V. For each voltage value, the value of electrical current is measured five times, and the mean value of these measurements is reported in correspondence with the voltage applied in the IV curve, from which the electrical conductivity was calculated via Ohm’s law. Electrical conductivity was measured on five samples for each filler concentration. Piezoresistive properties were investigated during the tensile test. Each sample has been tested three times. The mechanical response was monitored using an Instron dynamometer (series 5967, Instron, Norwood, MA, USA) equipped with a long-stroke extensometer (XL) with a 750 mm stroke to measure the actual sample strain. A 1 mm/min strain rate was applied to specimens (dimensions: 0.1 × 10 × 1 cm^3^). The variation in two-wire electrical resistance during the tensile test was measured using a Multimeter 3458A (Agilent, Santa Clara, CA, USA) in conjunction with a custom-designed LabVIEW program, which had an acquisition frequency of 2 Hz.

## 3. Results and Discussion

### 3.1. Electrical Properties

**Figure 1:**
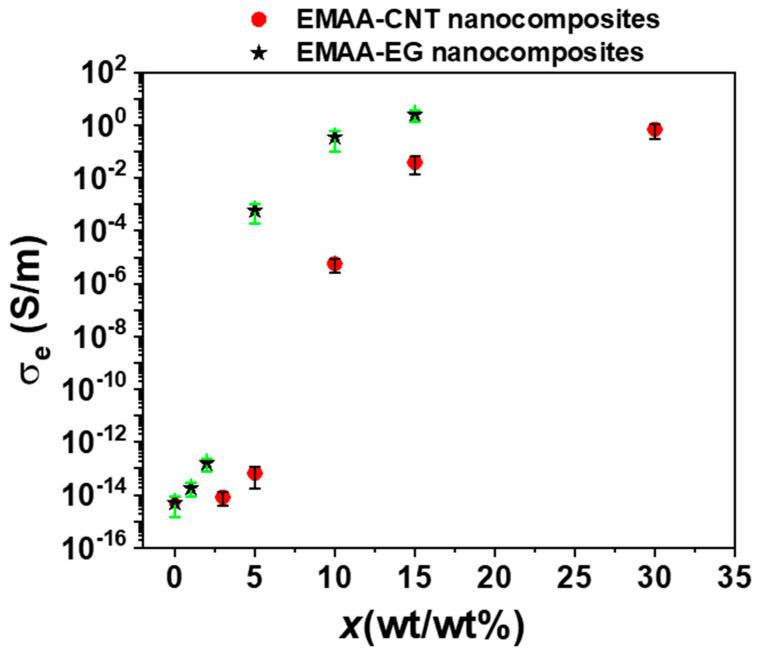
Electrical percolation thresholds of EMAA-CNT nanocomposites and EMAA-EG nanocomposites.

The electrical conductivity to the EMAA matrix has been imparted by adding CNTs and EG filler in different concentrations in such a way as to investigate the region near the electrical percolation threshold (EPT), which ranges from the point where the electrically conductive network starts to form until it is entirely built. Figure 1 shows the electrical conductivity (σe) as a function of the filler concentration (%wt./wt.). A considerable increase in electrical conductivity is obtained by adding conductive fillers in percentages equal to or higher than 5% for CNTs and 10% for EG.

The electrical conductivity passes from 10^−16^ S/m of the insulating polymeric matrix alone [40] to 2.60 ± 1.22 S/m for 15 wt% of CNT and 0.41 ± 0.19 S/m for 30 wt% of EG. The insulator-to-conductor transition happens only when a continuous conductive path allows the electron to flow through the material. According to the electrical percolation threshold theory, the inter-particle distances must be sufficiently near the so-called “tunneling distance” to allow an appreciable electrical current flow [41,42]. In the two cases studied herein, the insulator-to-conductor transition occurs below 5 wt% for CNT-based nanocomposites and below 10 wt% for EG-based nanocomposites. The difference in the EPT values is most likely attributable to the different number of functional groups on the edges of the two types of fillers and their shape [43]. With regard to the first aspect, based on the characterization of the nanofillers summarized in the Supplementary Materials, a higher content of oxygen was detected for EG than CNTs, meaning that on the edge surface of EG nanoparticles, there is a larger number of oxygenated functional groups, which reduces the electrical conductivity, increasing the electrical percolation threshold value.

Moreover, although both nanofillers show a high aspect ratio (approximately 1000), the EG is classified as a 2D particle, while a CNT is a 1D nanoparticle (second aspect). Concerning the first aspect, the elemental composition, evaluated through X-ray Photoelectron Spectroscopy (XPS), provides a value of the ratio C/O of 74.76 for the CNT filler and 35.10 for the EG (see Figure S1c and Figure S2c, respectively).

From transmission electron microscopy (TEM) images shown in the Supplementary Materials section, CNTs (see Figure S1d) appear as long and interconnected bundles like ropes, while EG nanoparticles (see Figure S2d) have a sheet-like shape. The rope structure of the CNT allows the formation of a conductive network at a lower concentration of the filler than that obtained for the 2D nanofiller; similar results were obtained with nanocomposites based on a thermosetting matrix [44]. Electrical results are corroborated by the FESEM investigation described in the following paragraph.

### 3.2. Morphological Investigation: FESEM Images

**Figure 2:**
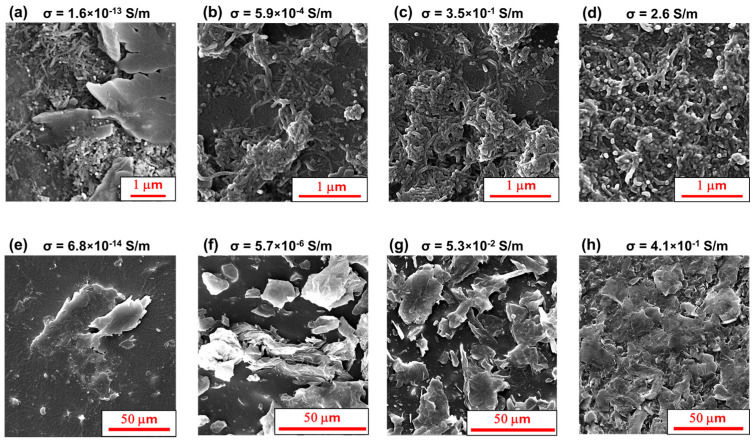
FESEM images of EMAA nanocomposites and their electrical conductivity values: (**a**) EMAA 2% CNT; (**b**) EMAA 5% CNT; (**c**) EMAA 10% CNT; (**d**) EMAA 15% CNT; (**e**) EMAA 5%EG; (**f**) EMAA 10% EG; (**g**) EMAA 15% EG; (**h**) EMAA 30% EG.

Figure 2 shows FESEM images of EMAA nanocomposites in correlation with their relative electrical conductivity values. An oxidizing treatment was performed on the samples before morphological investigation to make nanofiller particles more visible. The main aspect to be highlighted is the increase in the density of the CNTs (Figure 2a–d) and EG (Figure 2e–h) networks with increasing filler content from the EPT concentration to higher concentrations. In Figure 2a, the carbon nanotubes (CNTs) are interspersed between regions of pristine matrix. The conductive path is not completely formed, and only a few contacts exist between the CNTs. In Figure 2b,c, CNTs appear distributed over various areas of the matrix, yet without covering the entire investigated surface, thus justifying the lower conductivity of EMAA 5% CNT and EMAA 10% CNT with respect to the EMAA 15% CNT. In fact, for the sample EMAA 15% CNT, which contains the highest analyzed filler content and is well beyond the EPT value, the CNTs are distributed more densely, covering the entire analyzed surface (Figure 2d).

In the same way, according to the EPT of EG-based nanocomposites (Figure 1), at 5 wt%, EG particles are arranged in isolated blocks as shown in Figure 2e, while at 10 wt%, EG particles begin to be closer to form the conductive network (Figure 2f).

Further increases in the filler content reduce the insulating regions between EG particles, making them better interpenetrated into the matrix (Figure 2g,h).

Moreover, FESEM provides evidence on the role of filler shape in constructing the conductive path. The rope-like shape of CNTs allows for the easy creation of contact points between one CNT and its adjacent ones. In the case of EG filler, the sheet-like shape requires a higher concentration of filler content to build a continuous conductive path. This condition is evident when comparing Figure 2d with Figure 2g, where the matrix is loaded with the same filler concentration (i.e., 15% CNT and 15% EG, respectively). Insulating zones of the polymeric matrix are not detectable in the EMAA 15% CNT system.

In comparison, some EG particles appear sufficiently far apart in the EMAA 15% EG sample to hinder the electrical current flow. These results perfectly agree with those related to the thermogravimetric analysis commented on in the next section.

### 3.3. Thermal Analysis

#### 3.3.1. Thermogravimetric Analysis (TGA)

**Figure 3:**
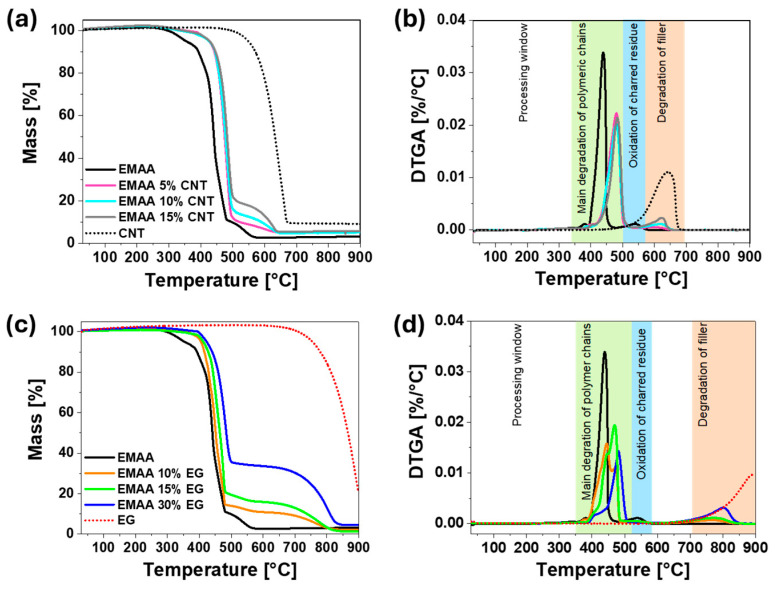
(**a**) TGA curves of EMAA-CNT nanocomposites, EMAA matrix, and CNT filler; (**b**) DTGA curves of EMAA-CNT nanocomposites, EMAA matrix, and CNT filler; (**c**) TGA curves of EMAA-EG nanocomposites, EMAA matrix, and EG filler; (**d**) DTGA curves of EMAA-EG nanocomposites, EMAA matrix, and EG filler.

Thermogravimetric analysis was carried out to evaluate the effect of the two typologies of nanofillers and their percentage on the thermal stability of the EMAA polymer, as previously performed with a thermosetting polymeric matrix. Figure 3 shows the TGA and derivative thermogravimetric curves (DTGA), obtained under reactive atmosphere (air), of the EMAA-CNT and EMAA-EG nanocomposites, together with the two nanofillers (CNTs and EG powder) and EMAA matrix, respectively.

As expected, CNTs and EG filler (dot lines in Figure 3a,c) are more thermally stable than the EMAA matrix alone (solid black line in Figure 3a,c). EG nanoparticles are stable up to 700 °C, about 100 °C higher than CNTs. The presence of the filler in the nanocomposites causes an increase in their thermal stability since filler nanoparticles hinder the thermo-oxidation of the polymer in the early stage [45]. Both thermogravimetric curves manifest two main steps of degradation events, the first due to the polymeric matrix and the second/last one due to filler degradation. The study of DTGA curves (Figure 3b,d) allows us to identify at least three main degradation steps after the interval of temperature where the nanocomposites are stable (i.e., processing windows), which are (i) the main degradation of polymer chains, (ii) the oxidation of charred residue, and (iii) the degradation of filler. The first degradation step is due to dehydration and random and homolytic scission of a methoxycarbonyl side group [33,46] followed by the degradation of the ethylene main chains [34]. The second step, at a temperature higher than 510 °C, is due to oxidation of the charred residue formed by oxidative dehydrogenation of EMAA, taking place throughout the heating process in air, besides thermal oxidation and pyrolysis (for samples with CNTs). In particular, the charred residue obtained immediately downstream from the first loss curve completely disappears in the second main degradation step corresponding to the filler degradation only, whose value strongly depends on the filler nature. Since EG filler degrades at a higher temperature than CNTs, the degradation step for EG in the nanocomposites starts at a higher temperature (around 700 °C) with respect to the value of 570 °C related to the nanocomposites with CNT filler. As expected, the EMAA component does not influence the degradation of the carbonaceous filler, whereas the filler considerably stabilizes the polymer at the beginning of the degradation phenomena.

The extension of the processing window changes from CNT-based nanocomposites to EG-based nanocomposites, even though it is only by a few degrees. More specifically, in Tables S1 and S2 of the Supplementary Materials, the value of the initial degradation temperature, evaluated at a 5% weight loss, is higher for all the experimented concentrations of CNT nanocomposites than for the EG nanocomposites. This result is most likely due to the type of network that hinders the diffusion of gases and favors heat dissipation within the sample. Filler particles physically hinder both the penetration of oxidative gases into the matrix and the release of pyrolytic gases (“labyrinth effect”) [47], increasing the average free path of gases. Simultaneously, the good thermal conductivity of CNTs and EG minimizes the heat retention in the material, further contributing to improving its thermal stability [48,49]. By increasing the filler content in nanocomposites, not only does the electrical conductivity increase but so does the thermal conductivity [50]. It follows that the denser the conductive network, the more the labyrinth effect and heat dissipation are guaranteed. For these reasons, the degradation temperature is expected to be higher in the nanocomposites where the electrical path is formed. As shown in paragraph 3.1, the electrically conductive path composed of CNT forms at a lower filler concentration than the EG filler. This aspect explains why EMAA-CNT nanocomposites at lower filler concentrations are more thermally stable than EMAA-EG nanocomposites. In other words, at the same filler content of 15 wt% in the EMAA-CNT sample, the conductive network is already formed, and it is the same network that protects the polymeric chains against the gas contacts and dissipates heat.

On the other hand, at 15 wt% of EG, the conductive network is not completely formed (as also highlighted in Figure 2g of the FESEM images). Thus, it is less effective against degradation. As well as the electrically conductive network that forms in the EMAA-EG nanocomposites at 30 wt%, the degradation temperature becomes more similar to that of the EMAA-CNT nanocomposite. The FESEM image (Figure 2h) of EMAA 30% EG confirms the presence of a more robust filler network against oxidative gas diffusion and heat retention.

Another aspect to consider is the shape of the main peak in the DTGA, which differs between EMAA-CNTs and EMAA-EG nanocomposites. In the case of CNT-based nanocomposites, carbon nanotubes form a thin layer of polyaromatic carbon char, protecting the underlying polymer from oxygen. For this reason, the thermal stability does not change significantly with the filler concentration, as confirmed by Bocchini et al. [45] in a study on polyethylene-based composite. For EG-based nanocomposites, it can be noted that step 1 becomes composed of two peaks, one near the peak of the pristine matrix and the other one at a higher temperature. The changes in the DTGA curves suggest that at 10% wt. of EG, a fraction of the matrix degrades almost at the same temperature as the unfilled matrix, whereas a second fraction degrades at a higher temperature (see the profile of the orange curve in Figure 3d). This behavior is mainly due to the morphological arrangement of the graphitic block in the matrix. Most probably, it is also possible to draw a correlation with the electrical behavior of the sample. In fact, in EMAA 10% EG, at a low concentration of the filler particles, there are still a few graphitic blocks in forming a percolative network. From an electrical point of view, the path of oxidative gases is not so tortuous, and they can easily reach some of the unprotected matrix regions. Then, the sample exhibits a fraction of the polymeric matrix that degrades at almost the same temperature as the unfilled matrix and a second fraction with a higher degradation temperature, corresponding to the fraction that degrades at higher temperatures (see the profile of the orange curve in Figure 3d). For higher filler concentrations, this phenomenon is less marked and the main change is observed at higher temperatures, as expected when the percolative network is completely formed as for the 30 wt% of the filler particles.

#### 3.3.2. Differential Scanning Calorimetry (DSC)

**Figure 4:**
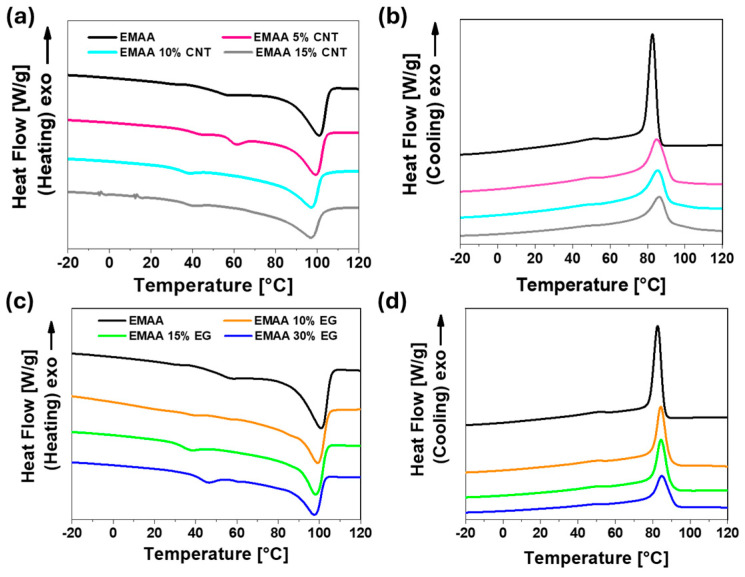
(**a**) Heating and (**b**) cooling curves of DSC of EMAA-CNT nanocomposites and EMAA matrix; (**c**) heating and (**d**) cooling curves of DSC of EMAA-EG nanocomposites and EMAA matrix.

Differential scanning calorimetry allows the determination of the crystallinity degree (X_c_), melting temperature (T_m_), crystallization temperature (T_c_), and glass transition temperature (T_g_), which are helpful in the investigation of the structure of the polymer, which strongly affects the mechanical properties of the material. The crystallinity degree (X_c_) of the EMAA and the composites was determined by the melting peak area as follows (Equation (1)):

(1) $$X_{c}=\frac{{∆H}_{m}}{{f*∆H}_{m}^{0}}$$

where ∆H_m_ is the melting enthalpy of the sample, ∆H^0^_m_ is the melting heat for 100% crystalline EMAA (taken to be 290.4 J/g, [51]), and *f* is the mass fraction of matrix in the nanocomposite materials. Figure 4 shows the DSC heating and subsequent cooling thermograms of the EMAA nanocomposites, while Table 2 and Table 3 summarize the corresponding thermal parameters.

Both the heating and cooling curves of EMAA nanocomposites suggest the formation of small crystals that originate from filler nanoparticles acting as heterogeneous nuclei. Heating curves in Figure 4a and c show that the melting temperature tends to decrease by a few degrees (see Table 2 and Table 3) with increasing filler content (both for CNTs and EG), indicating a reduction in crystal size. This hypothesis is also confirmed by the widening of the melting peak in the presence of filler nanoparticles, which opens at lower temperatures than that of the matrix alone. The nucleation effect of filler nanoparticles is also evident from the cooling curves of Figure 4b,d. In fact, the crystallization enthalpy increases with increasing filler content, and the crystallization event starts at higher temperatures both in the presence of CNTs or EG filler (see T_c_ values in Table 2 and Table 3). Upon increasing the filler content (from 5% to 10% for EMAA-CNT nanocomposites and from 10% to 15% EG for EMAA-EG nanocomposites), the number of nuclei increases, leading to an increase in small crystals and the crystallinity degree [52]. However, when the filler content is high, small crystals are not meant to grow as in the matrix alone because the arrangement of conductive filler in a network structure exerts a nanoconfinement effect. This means that, while the nucleation effect of the nanoparticles favors the formation of crystals, the nanoconfinement effect limits their growth. In the sample of EMAA 15% CNT, the nanoconfinement effect prevails over the nucleation effect, and the degree of crystallinity lowers.

### 3.4. Mechanical Characterization

#### 3.4.1. Dynamic Mechanical Analysis (DMA)

**Figure 5:**
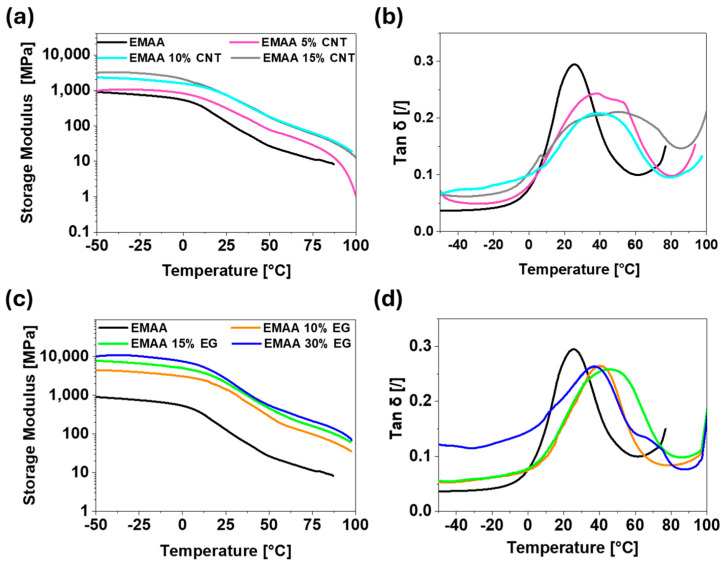
DMA results: (**a**) storage modulus and (**b**) tan δ of EMAA-CNT nanocomposites and EMAA matrix; (**c**) storage modulus; and (**d**) tan δ of EMAA-EG nanocomposites and EMAA matrix.

DMA analysis was performed to investigate the changes in the mechanical response of the matrix in the presence of the two different fillers. The storage modulus and loss factor (tan δ) vs. temperature for the neat polymer and nanocomposites are reported in Figure 5. The storage modulus begins to decrease from 0 °C to 80 °C (Figure 5a,b), where the glass transition event is observed, as indicated by the tan δ profile in Figure 5c,d. As expected, the introduction of rigid fillers (CNTs and EG) in the polymeric matrix causes an increase in storage modulus. This is because both the theoretical modules of CNTs (around 0.2–1.0 TPa [53]) and EG (around 1.0–2.8 TPa [54,55]) are higher than that of the matrix. By comparing the storage modulus of EMAA-CNT nanocomposites with that of EG-based nanocomposites (Figure 5a,c), a higher increase is detected in the presence of EG filler. A higher number of polar groups on the surface of the 2D filler (see Supplementary Materials), together with a higher surface area of EG, leads to better filler–matrix interactions responsible for an effective transmission of load applied from the matrix to the more rigid filler.

The tan δ profile also changes due to the presence of the filler (Figure 5b,d).

According to the theory of Stimonaris et al. [56], the tan δ profile can be analyzed as a distribution of multiple relaxation phenomena involving regions with different chain mobility. In the case of nanocomposite materials, filler nanoparticles represent physical knots in the matrix, limiting chain movements and causing a shift in the maximum of tan δ profiles of EMAA nanocomposites at higher temperatures [57]. The shift of the tan δ peak means that in the nanocomposites, the fraction of regions at reduced mobility increases. In fact, polymeric chains confined between filler nanoparticles have low freedom of movement due to both nanoparticle–polymer interactions and filler rigidity.

The increase in the crystallinity obtained by introducing the filler (see Section 3.3.2), added to the contribution of polymeric chains confined between filler nanoparticles, causes a shift and an enlargement of the tan δ peak.

This last phenomenon is particularly relevant for EMAA 15% CNT and EMAA 30% EG samples. In these samples, the conductive network is more “robust” since the higher filler content implies a dense dispersion of nanoparticles in the whole matrix, as confirmed by electrical and morphological investigation. In these two cases, the contribution of regions characterized by nanoparticle–polymer interactions is significant.

#### 3.4.2. Tensile Test

The real mechanical deformation, here also indicated as true strain, is the deformation of the material calculated by considering the necking phenomenon. The EMAA matrix is a soft material that undergoes cross-section reduction during the loading phase (i.e., the necking phenomenon). For this reason, from a mechanical point of view, it is more appropriate to convert the stress and the strain calculated on the base of the initial cross section of the sample, σ and ε, respectively, into the true stress and the true strain (i.e., $\sigma_{r}$ and $\varepsilon_{r}$, respectively), considering the cross-section reduction in the materials during the loading phase as follows:

(2) $$\varepsilon_{r}=\ln\left(\varepsilon +1\right)$$

(3) $$\sigma_{r}=\sigma \cdot (\varepsilon +1)$$

**Figure 6:**
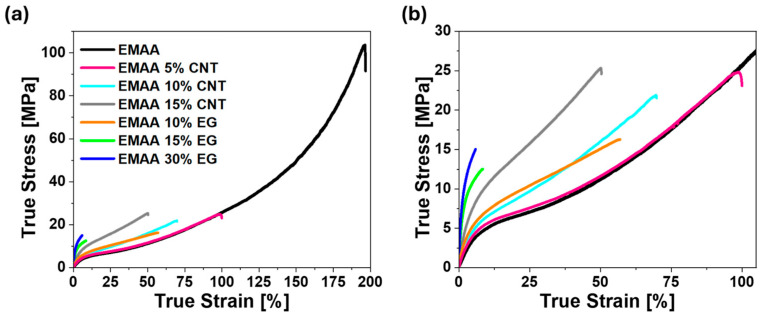
(**a**) Mechanical curve of the EMAA matrix and its nanocomposites; (**b**) enlargement at lower strains.

The comparison of the mechanical properties of EMAA pristine matrix with those of nanocomposite systems is reported in Figure 6.

The mechanical curve of the EMAA matrix is typical of a block copolymer [53], characterized by two distinct regions: region 1, below 10% of true strain, and region 2, above this point, up to failure. Region 1 includes the short elastic region and the beginning of plastic deformation. In fact, for soft polymers, the elastic region is restricted to low strains. Due to their organization into soft and rigid domains, block copolymers do not exhibit an evident yield stress [54]. The passage from region 1 to region 2 is characterized by a reduction in the slope of the mechanical curve. This means that the strain increases faster than the applied stress due to the propagation of the necking phenomenon throughout the entire sample. In region 2, the stress increases almost linearly with the strain, absorbing energy to align the macromolecules in the direction of the load. The enlargement in Figure 6b evidences the similarity between the mechanical curve of EMAA-CNT nanocomposites with that of the pristine matrix alone and, at the same time, the limitation of the EMAA 15% EG and EMAA 30% EG curves in the first region. The two fillers in the matrix differently affect the behavior of the stress–strain curve. In the case of CNT-based polymer, thanks to their 1D shape and high aspect ratio, the CNTs can easily interpenetrate macromolecules and accompany their movement under the applied load in the elastic region. At low CNT content, the curve of EMAA 5% CNT remains very similar to that of the EMAA matrix. As for electrical properties, in this case, the effect of the filler is still limited due to its low content. As filler concentration increases, the Young modulus of EMAA 10% CNT increases by 145% with respect to the matrix alone due to the efficient load transfer from the soft matrix to the more rigid CNTs. The functional groups of the matrix, in fact, could interact with the groups on the surface of the CNTs [58], whose presence is demonstrated by the oxygen content detected in the XPS analysis (see Figure S1c). In addition, the high aspect ratio of the filler causes adequate stress transmission via interfacial shear between the CNTs and the polymer [59]. CNTs continue to untangle, deforming together with the matrix until the applied load overcomes a critical value that causes CNT debonding from the matrix (“stick-slip” phenomenon [60]). From this point on, in region 2, macromolecules can straighten, as in pristine EMAA, due to the loss of stress transfer between the nanotubes and the matrix.

On the contrary, the mechanical curve does not exhibit a second linear region in the cases of EMAA 15% EG and EMAA 30% EG. According to the XPS results (Figure S2c), the oxygen content is higher than that of CNTs (Figure S1c). It follows that a higher number of functional groups decorates the surface of EG nanosheets. This aspect justifies both the higher Young modulus and the absence of region 2. The higher the number of interactions between the filler nanoparticles and the matrix, the better the load transfer from the soft matrix to the rigid EG nanosheets (i.e., improvement in Young modulus). Moreover, when high stresses are applied to the material above the elastic regime, the material directly undergoes failure. The trend of the mechanical curve of EMAA 10% EG also evidences the absence of the stick-slip phenomenon in EG nanocomposites since the higher filler–matrix interactions do not allow the matrix to disengage from the filler before failure, as happens on the converse for EMAA 10% CNT.

### 3.5. Piezoresistive Response

This paragraph compares the piezoresistive responses of CNT- and EG-based nanocomposites. The piezoresistive behavior of the two systems has been obtained by combining the mechanical test with the electrical resistance variation. Among all the experimented concentrations, EMAA 10% CNT and EMAA 15% EG have been selected as these samples show an electric conductivity value suitable for self-sensing applications. In the case of low electrically conductive samples (i.e., EMAA 5% CNT and EMAA 10% EG), the reproducibility of the electrical response is low, while in the case of highly concentrated samples (EMAA 15% CNT and EMAA 30% EG), the sensitivity to the strain is reduced (as reported in the Supplementary Materials) [61]. Near the EPT concentration, a nanocomposite system exhibits high sensitivity to strain due to the lower robustness of the conductive network, such that a very small deformation is sufficient to cause a variation in electrical resistance. However, although high sensitivity is desired in the application fields (i.e., structural health monitoring), handling sensors sensitive to small deformations would become difficult because there would be a risk of modifying their piezoresistive response already during the integration phase in the composite structure, for example. It is what happens when several tensile tests are performed on nanocomposites at the EPT concentration. The error in the material’s sensitivity is so high that the sensor’s trustworthiness is compromised. Moreover, a filler concentration higher than the EPT value ensures a better linearity of nanocomposite sensors compared to low-concentration materials. This is a strategy already adopted in the literature for soft matrix, like EMAA, whose elastic region in the absence of rigid fillers covers a restricted range of deformations [62]. In summary, EMAA 10% CNT and EMAA 15% EG are the best compromise for guaranteeing high strain sensitivity and good linearity in strain sensors made of a soft polymeric matrix.

The sensitivity to the mechanical strain is calculated in terms of the gauge factor (G.F.) defined as follows:

G.F.=∆RR0ℇr

where ∆R is the electrical resistance variation with respect to the initial resistance value $R_{0}$ and ${ℇ}_{r}$ is the real mechanical deformation of the sample. The so-defined gauge factor is the equivalent of the slope of the piezoresistive curve in the elastic regime. A high G.F. value suggests that small deformations can be detected by high electrical resistance.

**Figure 7:**
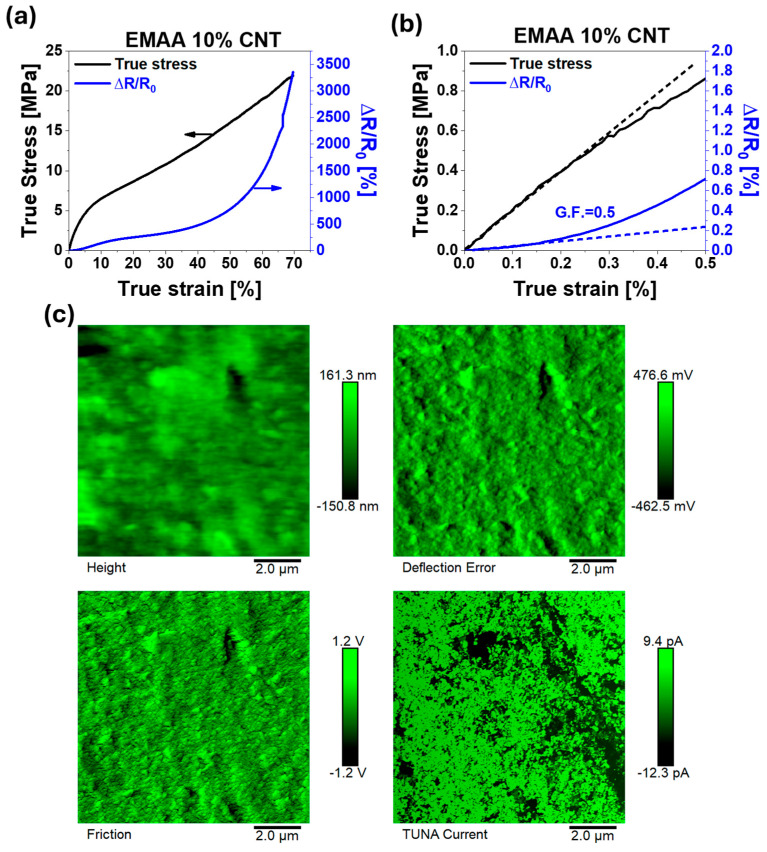
(**a**) Piezoresistive response during the tensile test; (**b**) enlargement of the piezoresistive curve; (**c**) TUNA morphology of EMAA 10% CNT.

**Figure 8:**
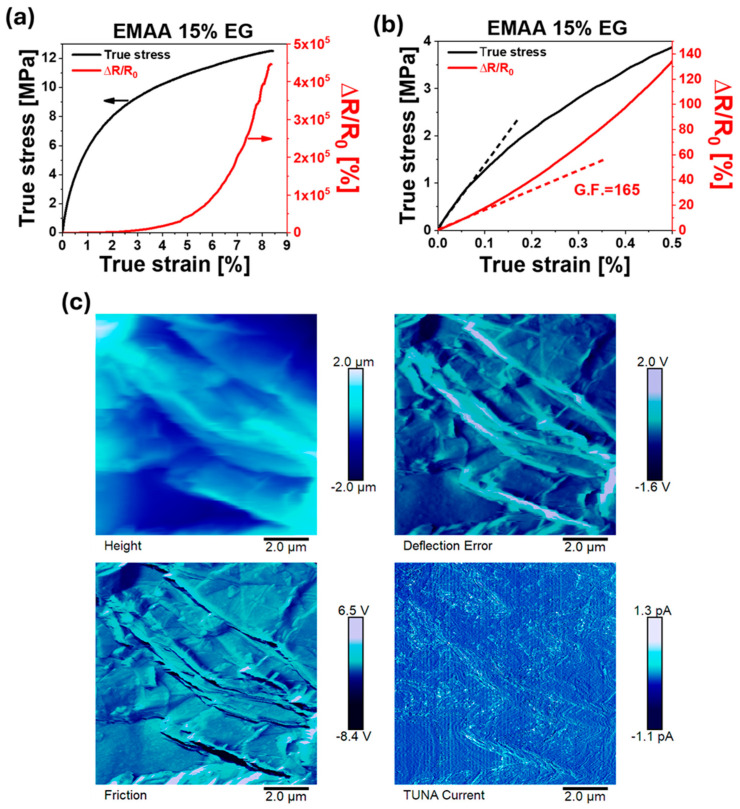
(**a**) Piezoresistive response during tensile test; (**b**) enlargement of piezoresistive curve; (**c**) TUNA morphology of EMAA 15% EG.

In Figure 7 and Figure 8, the mechanical curves of selected nanocomposite systems are presented together with their piezoresistive response (Figure 7a and Figure 8a).

To fully understand the differences obtained in the piezoresistive results, TUNA images of the two systems (EMAA 10% CNT and EMAA 15% EG) are reported in Figure 7c and Figure 8c, respectively.

The first aspect emerging from Figure 7a and Figure 8a is that the electrical resistance variation closely follows the mechanical trend, both in the case of EMAA 10% CNT and EMAA 15% EG. This result is also confirmed at high filler concentrations (see Figure S3a,b of the Supplementary Materials). In the case of EMAA 10% CNT, the piezoresistive response can be divided into two consecutive exponential trends, corresponding to linear trends on the mechanical curve of regions 1 and 2, respectively. On the other hand, EMAA 15% EG, characterized by a short elongation at break limited to region 1, has a piezoresistive response consisting of a single exponential trend. The exponential trend is typical of the “tunnelling effect”, according to which electrons can pass from one conductive particle to the adjacent one only if the inter-particle space is sufficiently close to the so-called “tunneling distance” [63,64]. This distance may change with filler size and concentration, interphase thickness, and surface energy [65]. When the nanocomposite material is under a tensile stress, the electrical resistance at the interface between particles (i.e., tunnelling resistance) increases because of a variation in the tunneling distance and contact area, causing a monotonic increase in the electrical resistance of the whole sample.

The difference emerging from the piezoresistive curves of EMAA-CNTs and EMAA-EG nanocomposites (Figure 7a, Figure 8a and Figure S3) demonstrates that the piezoresistive response does not depend only on the interruption of electrical contact when the nanocomposite is under strain but it is also strongly affected by the chemical interactions between the filler and the matrix. Most of the papers in the literature where CNTs have been added to a soft thermoplastic matrix report a piezoresistive response characterized by a single exponential trend [62,66,67]. The transition from the first exponential step to the second one in EMAA 10% CNT corresponds to the CNT debonding from the matrix (“stick-slip” phenomenon) described in the previous paragraph. The lower filler–matrix interactions in the case of EMAA-CNT samples modify not only the mechanical curve but also the piezoresistive response. On the contrary, when the filler–matrix interactions are stronger, as in the case of EMAA-EG nanocomposites, the typical single-exponential trend is obtained.

The G.F. of EMAA 10% CNT is 0.5 ± 0.08 (see Figure 7b), while a much higher value is obtained in the case of EMAA 15% EG, which is 165 ± 14 (see Figure 8b). Not only in the linear region but along the entire tensile test does the EG-based nanocomposite remains more sensitive to the strain than CNT nanocomposites, as can be noted by the right vertical axis scale of Figure 7a and Figure 8a. The G.F. values obtained for both nanocomposites are in line with or even better than other results in the literature for similar matrices and fillers. In more detail, in the presence of CNTs, the G.F. is between 1 and 2 when the filler content is 8% by weight [68]. Considering the slightly higher filler concentration in EMAA 10% CNT, the obtained value of G.F. is in line with those already reported in the literature. It is worth noting that the value of 0.5 is calculated in the mechanical elastic regime up to a strain value of 0.15%. Above the elastic limit, the slope of the piezoresistive response (sensitivity to the strain) becomes 18.3. The G.F. of EMAA 15% EG is surprisingly higher than the values reported in the literature [68,69], especially if the high filler content is considered. This result allows for the combination of a high piezoresistive response with better mechanical performance. Moreover, based on the present discussion, the type of filler for a self-sensing system can be appropriately chosen according to the application field. EMAA-CNT nanocomposites with high elongation at break and a lower sensitivity to the strain are suitable for movement sensors in the soft robotics field (such as for prosthesis) where high deformation has to be detected [70]. On the other hand, the high sensitivity at low strain of EMAA-EG systems makes them convenient for structural health monitoring integrated sensors in more rigid composite structures, such as in aeronautical and automotive components, or wind turbines [71].

The reversibility of the piezoresistive response has also been investigated to detect the presence of residual plastic deformation in the material when the applied load is released. Considering the mechanical curves of Figure 7a and Figure 8a, which do not show an evident yield stress, piezoresistive reversibility was studied via multiple creep/recovery tests at low stress (1 MPa) on EMAA 10% CNT and EMAA 15% EG (see Figure S4 of the Supplementary Materials). A value of 1 MPa is a stress value just above the elastic region; thus, only small residual strains can occur in the material when it returns to rest. Figure S4 illustrates the mechanical curve in conjunction with the piezoresistive response. It is evident that mechanical hysteresis is accurately monitored by electrical resistance variation in both EMAA 10% CNT and EMAA 15% EG, indicating that their strain sensitivity is reliable even over repeated loading-unloading creep cycles. As expected, even during the reversibility tests, the piezoresistive response of EMAA 15% EG is higher than the ΔR/R_0_ of EMAA 10% CNT at the same strain value, as already found during the tensile test.

The higher sensitivity of the EG sample compared to that obtained with the CNT filler relies on how the different aspect ratios and particle contact areas affect the conductive network when the nanocomposite material is under load. To better understand this aspect, TUNA images in Figure 7c and Figure 8c show CNT- and EG-based conductive networks at a nanoscale level. TUNA images (Height, Deflection Error, Friction, and TUNA Current) reported in Figure 7c and Figure 8c were collected in a single region of the EMAA 10% CNT and EMAA 15% EG samples, respectively. From the Deflection Error image, the presence of carbon nanotubes can be detected as extended bundles along their length over the entire investigated area, as confirmed by the FESEM image at high magnification in Figure 2c. The Friction image serves as a map of the lateral flexion of the cantilever during the sample scan. This signal not only contains information about the friction between the sample and the tip but also offers topographic details about the non-flat sample surface. In the present case, the regions with a higher friction value correspond to the highly conductive areas that are more densely populated by CNTs. The conductive network composed of CNTs emerges more clearly from the TUNA Current image, for which electric current values ranging from −12.3 pA to 9.4 pA are measured. In the TUNA Current image, the electric current values associated with the local conductive domains dispersed throughout the matrix at the nanometric level are represented by the respective colors on the side scale bar. It is possible to distinguish a densely interconnected network characterized by a linked tangle arrangement, colored light green, in the entire investigated region. When external stress acts on the nanocomposites, the material deformation leads to an increase in the tunneling distance between CNT particles. However, since CNTs form an intricate network, new contacts may form with neighboring CNTs [72], thereby limiting a significant increase in electrical resistance.

In the case of EMAA 15% EG, the TUNA image, together with Friction and Deflection Error images, clearly shows partially overlapped EG sheets. When external stress is applied to EG-based nanocomposites, graphitic blocks change their arrangement and orientation, causing a reduction in the overlapping area and interlayer distance [72]. Due to their 2D structure, each EG particle may have a large contact area with another EG block. However, small deformation in the nanocomposite is sufficient to reduce the contact area, i.e., tunneling area, resulting in a significant variation in the electrical resistance [44]. For this reason, the increase in the electrical resistance sensed by the CNT nanocomposite is lower than the electrical resistance variation shown by the EG nanocomposite [73,74]. As can be seen from the TUNA Current image of the sample EMAA 15% EG, electric current values ranging from −1.1 pA to 1.3 pA are measured.

Based on the results obtained in the present study and a literature review, a comparison between CNTs and EG in terms of electrical properties, dispersion, mechanical performance, cost, processability, and environmental impact is reported in Table 4.

The choice between one filler and another may mainly depend on the application field’s requirements, such as high sensitivity to small or large deformations, low or high elongation at break, and cost limitations on raw materials and production processes.

## 4. Conclusions

This study addresses the lack of studies on the EMAA-EG piezoresistive response and its comparison with EMAA-CNT nanocomposites. The results summarized below can serve as a general guide for understanding the effects of two different nanometric fillers, CNTs and EG (1D and 2D, respectively), on the electrical, thermal, mechanical, morphological, and piezoresistive properties of thermoplastic nanocomposites. Moreover, at the same time, the obtained results provide the reader with a clear indication regarding the choice of a self-sensing system suitable for the final application (i.e., biomedical, automotive, and aeronautical).

From an electrical point of view, higher filler content is required in the case of EG particles, mainly due to the characteristic 2D shape, to reach the EPT. On the contrary, the rope-like shape of CNTs (1D filler) allows for reaching the EPT at lower filler concentrations.

CNTs seem to be more effective in the improvement in thermal stability than EG filler, since, as for the electrical percolation threshold, lower concentrations of CNTs are sufficient to create an interconnected protective network against oxidative gases.

Comparing the DSC results of EG-based nanocomposites with those of CNT-based nanocomposites, it can be concluded that both fillers have the same effect on the crystallinity of the EMAA matrix, slightly favoring the crystallinity.

DMA results of EG-based nanocomposites and CNT-based nanocomposites show a high elastic modulus in the case of the EG filler. The higher number of interaction between the EMAA matrix and the polar groups on the EG edges lead to a more effective transmission of the load applied from the matrix to the rigid filler in the EMAA-EG nanocomposites than in the EMAA-CNT nanocomposites.

A higher Young modulus of EG nanocomposites than CNT nanocomposites has been confirmed by tensile test. The lower number of CNT–matrix interactions favors the stick-slip phenomenon, resulting in high elongation at break, whereas EMAA-EG samples undergo direct failure at high applied stress.

Both the piezoresistive response of EMAA 10% CNTs and EMAA 15% EG perfectly reproduce the mechanical behavior during tensile tests. The 2D shape of EG nanoparticles justifies the much higher gauge factor of EMAA 15% EG (165 ± 14) than the value of EMAA 10% CNT gauge factor (0.5 ± 0.08).

## Figures and Tables

**Table 1:** Equipment and methods of TGA, DSC, and DMA analysis.

Analysis	Device	Method
TGA	Mettler TGA/SDTA 851 (Mettler-Toledo Columbus, OH, USA)	Temperature range from 25 °C to 900 °C, in air, heating rate 10 °C/min
DSC	Mettler DSC 822/400 (Mettler-Toledo Columbus, OH, USA)	Temperature range from −20 °C to 115 °C, heating and following cooling scan at 10 °C/min
DMA	DMA 2980 TA instrument (New Castle, DE 19720, USA)	Tensile mode at 1 Hz frequency, from −60 °C to 105 °C, at 3 °C/min
FESEM	SEM LEO 1525 (Carl Zeiss SMT AG, Oberkochen, Germany)	All samples were placed on a carbon tab previously stuck to an aluminum stub and then covered with a 250 Å thick gold film using a sputter coater (Agar mod. 108 A—Agar Scientific, Stansted, UK)
AFM-TUNA	NanoScope Analysis 1.80 (Build R1.126200; Bruker, Billerica, MA, USA)	The optimized parameters for this technique are reported in Table S1 of the Supplementary Materials

**Table 2:** Summary of DSC results of EMAA-CNT nanocomposites and EMAA matrix.

Sample	CNT (%)	∆H_m_ (J/g)	∆H_c_ (J/g)	T_m_ (°C)	T_c_ (°C)	X_c_ (%)
EMAA	0	43.0	54.9	102.1	82.2	14.8
EMAA 5% CNT	5	51.3	49.9	100.2	85.0	17.7
EMAA 10% CNT	10	51.1	52.7	97.05	85.4	17.6
EMAA 15% CNT	15	41.6	43.1	97.99	86.6	14.3

**Table 3:** Summary of DSC results of EMAA-EG nanocomposites and EMAA matrix.

Sample	EG (%)	∆H_m_ (J/g)	∆H_c_ (J/g)	T_m_ (°C)	T_c_ (°C)	X_c_ (%)
EMAA	0	43.0	54.9	102.1	82.2	14.7
EMAA 10% EG	10	53.5	57.0	100.1	84.4	18.3
EMAA 15% EG	15	52.5	57.4	98.19	84.4	17.9
EMAA 30% EG	30	51.4	53.1	98.66	84.9	17.6

**Table 4:** Comparison between CNTs and EG in terms of electrical properties, dispersion, mechanical performance, cost, processability, and environmental impact.

Property	Carbon Nanotubes (CNTs)	Expanded Graphite (EG)	Ref.
Electrical properties	High conductivity at lower loadings due to rope-like shape. Lower sensitivity strain than EG at the same strain.	Higher loadings to achieve similar CNT conductivity due to the sheet-like shape. Higher strain sensitivity than CNTs at the same strain.	[75,76,77]
Dispersion	Challenging due to agglomeration, CNT dispersion requires functionalization or the use of surfactants.	Easier exfoliation, but high loadings can lead to phase separation (aggregation).	[76,78,79]
Mechanical performance	Enhancement of tensile strength and elongation at break.	Reduction in flexibility and brittleness increase at high content.	[78,79,80]
Cost	High cost, due to complex synthesis and purification.	Low cost, derived from natural graphite.	[77,78]
Processability	Moderate impact on viscosity; manageable at low loadings.	High loadings increase viscosity, complicating melt processing.	[76,78,79]
Environmental Impact	Potential toxicity and environmental persistence.	Generally safer, though chemical exfoliation may have environmental concerns.	[78,80,81]

## Data Availability

The data presented in this study are available on request from the corresponding author due to privacy reasons.

## Supplementary Materials

The following supporting information can be downloaded at https://www.mdpi.com/article/10.3390/nano15130994/s1: Figure S1. (a) C1s scans of powders by XPS analysis; (b) O1s scans of powder by XPS analysis; (c) elemental composition and percentage of groups of CNTs by XPS analysis; (d) TEM image of CNTs; Figure S2. (a) C1s scans of powders by XPS analysis; (b) O1s scans of powder by XPS analysis; (c) elemental composition and percentage of groups of EG by XPS analysis; (d) TEM image of EG.; Figure S3. Piezoresistive response during tensile test of (a) EMAA 15% CNT and (b) EMAA 15% EG.; Figure S4. Reversibility of piezoresistive response: multiple creep/recovery tests at low stress (1 MPa) on (a) EMAA 10% CNT and (b) EMAA 15% EG.; Table S1. Characteristic temperatures of D-TGA of EMAA-CNT nanocomposites and EMAA matrix.; Table S2: Characteristic temperatures of D-TGA of EMAA-EG nanocomposites and EMAA matrix.
