# Supplementary material for: Thermal, Mechanical, Morphological, and Piezoresistive Properties of Poly(ethylene-co-methacrylic acid) (EMAA) with Carbon Nanotubes and Expanded Graphite
Source: Nanomaterials (Basel). 2025 Jun 26;15(13):994. doi: 10.3390/nano15130994 (PMC12250855; doi:10.3390/nano15130994)
Supplement: Supplementary file 1 [file nanomaterials-15-00994-s001.zip › nanomaterials-3648534-supplementary.pdf]

nanomaterials

MDPI

Supplementary materials

Thermal, Mechanical, Morphological, and Piezoresistive Properties of Poly(ethylene-co-methacrylic acid) (EMAA) with Carbon Nanotubes and Expanded Graphite

Francesca Aliberti [1,*], Luigi Vertuccio [2], Raffaele Longo [1], Andrea Sorrentino [3], Roberto Pantani [1], Liberata Guadagno [1] and Marialuigia Raimondo [1,*]

[1] Department of Industrial Engineering, University of Salerno, Via Giovanni Paolo II, 84084 Fisciano, Italy; rlongo@unisa.it (R.L.); rpantani@unisa.it (R.P.); lguadagno@unisa.it (L.G.)
[2] Department of Engineering, University of Campania "Luigi Vanvitelli", Via Roma 29, 81031 Aversa, Italy; luigi.vertuccio@unicampania.it
[3] Institute for Polymers, Composites, and Biomaterials (IPCB-CNR), Via Previati n. 1/E, 23900 Lecco, Italy; andrea.sorrentino@cnr.it
* Correspondence: faliberti@unisa.it (F.A.); mraimondo@unisa.it (M.R.)

Academic Editor(s): Ana M. Benito,Tomonori Ohba
Received: 1 May 2025
Revised: 10 June 2025
Accepted: 23 June 2025
Published: date

Citation: Aliberti, F.; Vertuccio, L.; Longo, R.; Sorrentino, A.; Pantani, R.; Guadagno, L.; Raimondo, M. Thermal, Mechanical, Morphological, and Piezoresistive Properties of Poly(ethylene-co-methacrylic acid) (EMAA) with Carbon Nanotubes and Expanded Graphite. *Nanomaterials* **2025**, *15*, x. https://doi.org/10.3390/xxxxx

Copyright: © 2025 by the authors. Submitted for possible open access publication under the terms and conditions of the Creative Commons Attribution (CC BY) license (https://creativecommons.org/licenses/by/4.0/).

1. Characterization of CNT and EG filler

All X-ray Photoelectron Spectroscopy (XPS) spectra were collected using Al–K radiation (1486.6 eV), monochromatized by a twin crystal monochromator, yielding a focused X-ray spot (elliptical in shape with a major axis length of 400 µm) at 3 mA × 12 kV. The alpha hemispherical analyzer was operated in the constant energy mode with survey scan pass energies of 200 eV to measure the whole energy band and 50 eV in a narrow scan to selectively measure the elements. XPS data were analyzed with Advantage software. A smart background function was used to approximate the experimental backgrounds, and surface elemental compositions were calculated from background-subtracted peak areas.

XPS results on CNT filler are shown in **Figures S1** a,b, while the obtained elemental composition and percentage of groups of CNT is summarized in **Figure S1** c.

XPS results on EG filler are shown in **Figures S2** a,b, while the obtained elemental composition and percentage of groups of EG is summarized in **Figure S2** c.

Transmission electron microscopy (TEM) investigation was carried out using a microscope JEOL model JEM-1400 Plus provided with an image acquisition camera model GATAN (ORIUS), and a resolution of 0,38 nm between dots and 0,2 nm between lines. The samples of EG and CNT filler powders were sonicated before the morphological analysis. As for SEM analysis on the nanofiller alone, a drop of each suspension was placed on the microscope grip and left to dry before TEM investigation. TEM images of CNT and EG are shown in **Figure S1** d and **Figure S2** d, respectively.

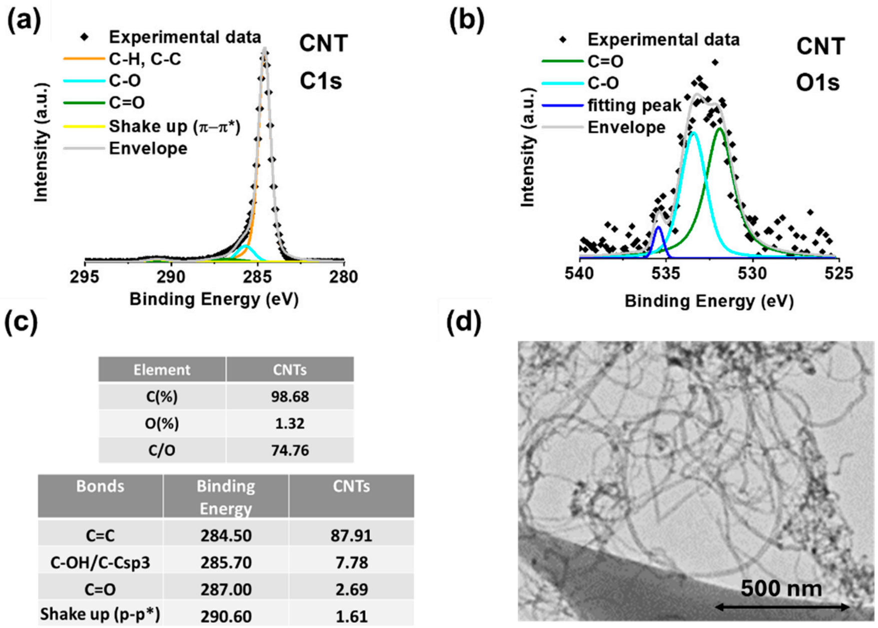

Element	CNTs
C(%)	98.68
O(%)	1.32
C/O	74.76

Bonds	Binding Energy	CNTs
C=C	284.50	87.91
C-OH/C-Csp3	285.70	7.78
C=O	287.00	2.69
Shake up (p-p*)	290.60	1.61

Figure S1. (a) C1s scans of powders by XPS analysis; (b) O1s scans of powder by XPS analysis; (c) elemental composition and percentage of groups of CNT by XPS analysis; (d) TEM image of CNT.

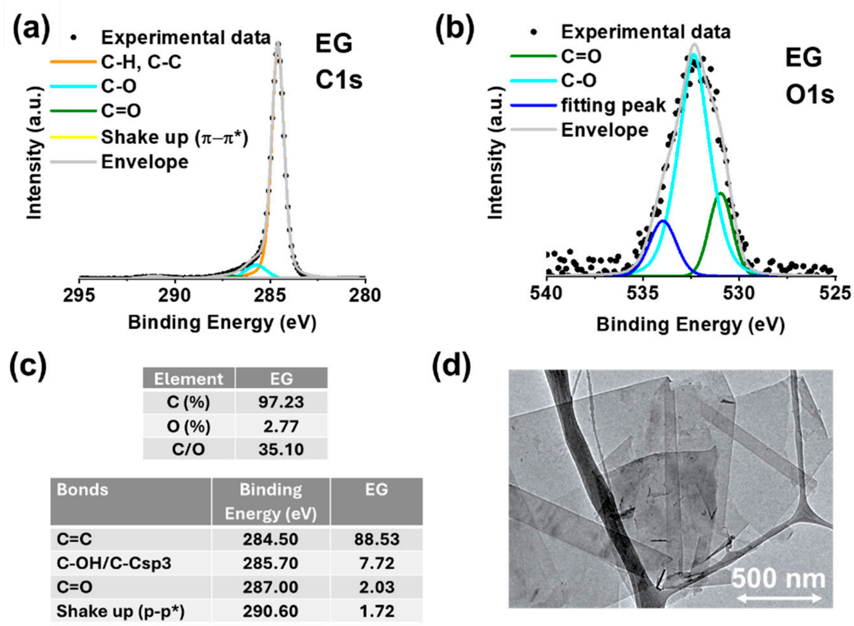

Element	EG
C (%)	97.23
O (%)	2.77
C/O	35.10

Bonds	Binding Energy (eV)	EG
C=C	284.50	88.53
C-OH/C-Csp3	285.70	7.72
C=O	287.00	2.03
Shake up (p-p*)	290.60	1.72

Figure S2. (a) C1s scans of powders by XPS analysis; (b) O1s scans of powder by XPS analysis; (c) elemental composition and percentage of groups of EG by XPS analysis; (d) TEM image of EG.

2. Thermogravimetric analysis (TGA)

Table S1. Characteristic temperatures of D-TGA of EMAA-CNT nanocomposites and EMAA matrix.

**Sample**	**CNT (%)**	**T_{5wt} (°C)**	**T_{50wt} (°C)**
**EMAA**	0	401.9	438.6
**EMAA 5%**	5	426.0	474.7
**EMAA 10%**	10	426.4	476.9
**EMAA 15%**	15	427.6	482.8

Table S2. Characteristic temperatures of D-TGA of EMAA-EG nanocomposites and EMAA matrix.

**Sample**	**EG (%)**	**T_{5wt} (°C)**	**T_{50wt} (°C)**
**EMAA**	0	401.9	438.6
**EMAA 10% EG**	10	405.2	451.9
**EMAA 15% EG**	15	412.5	464.7
**EMAA 30% EG**	30	423.1	484.5

1. Piezoresistive response

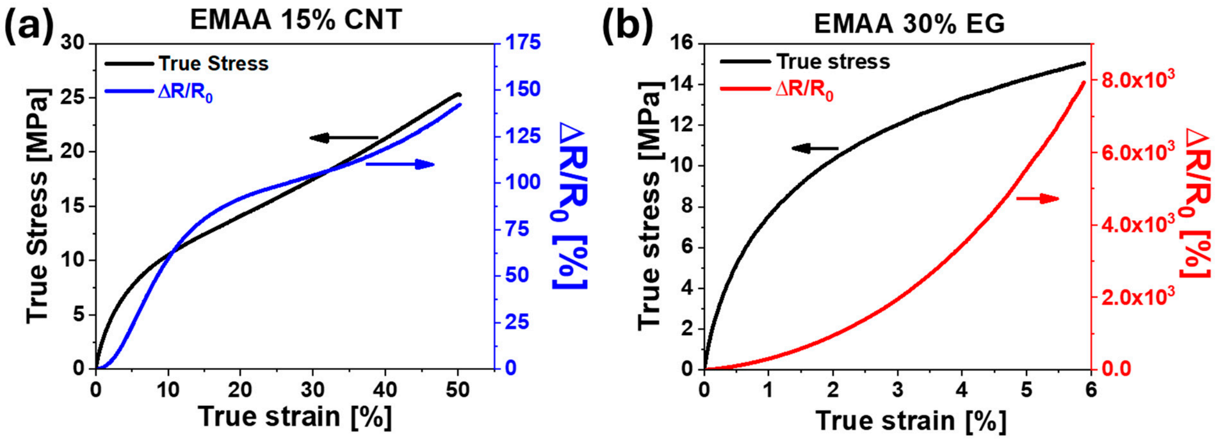

Figure S3. Piezoresistive response during tensile test of a) EMAA 15 % CNT and b) EMAA 15 % EG.
